# Prediction of Blood Transfusion Need and Dose in Patients With Upper Gastrointestinal Bleeding: Retrospective Multicenter Prediction Model Study

**DOI:** 10.2196/83889

**Published:** 2026-08-04

**Authors:** Xiaoyu Li, Yuqin He, Mingyang Hou, Zhi Yu, Shuai Miao, Zhiyong Huang, Min Yang

**Affiliations:** 1 School of Microelectronics and Communication Engineering, Chongqing University, No. 174 Shazheng Street, Shapingba District, Chongqing, 400044, China, 86 023-65678860; 2 Bioengineering College of Chongqing University, Chongqing University, Chongqing, 400044, China; 3 Department of Gastroenterology, Daping Hospital, Army Medical Center of PLA, Army Medical University, Chongqing, 400042, China

**Keywords:** blood transfusion, decision support systems, clinical, electronic health records, gastrointestinal hemorrhage, machine learning, regression analysis, ROC curve, receiver operating characteristic

## Abstract

**Background:**

Transfusion thresholds in upper gastrointestinal bleeding are debated; hemoglobin cutoffs of 70‐80 g/L are widely cited yet inconsistently applied. Common risk scores offer limited individualized guidance and rarely provide calibrated, interpretable predictions for transfusion decisions.

**Objective:**

This study aimed to develop and validate a two-stage, clinically constrained gradient-boosting framework (Medically Constrained Gradient Boosting [MCGB]) that predicts transfusion need and estimates transfusion dose with quantified uncertainty and to implement a prototype recommendation system for clinical use.

**Methods:**

We analyzed a retrospective multicenter cohort of 849 adults with endoscopically confirmed upper gastrointestinal bleeding admitted to 3 hospitals in Chongqing, China (January 2019 to August 2025). Predictors available before the transfusion decision included demographics, first recorded vital signs, initial laboratory indices, and clinician-adjudicated etiology. Stage 1 used a calibrated classifier with prespecified monotonic constraints and stability-screened, clinically justified interactions. Stage 2 modeled transfusion dose via quantile predictions with conformal adjustment to generate 95% prediction intervals. Performance was assessed using a cross-site hold-out design. Overall, 2 hospitals were used as the development cohort, within which stratified 5-fold cross-validation was performed for model development, hyperparameter tuning, interaction screening, and calibration. The remaining hospital was held out as an independent test cohort for final evaluation. Hospital-wise alternating external testing was further conducted as a supplementary robustness analysis to assess performance stability across institutions. Classification performance was evaluated using discrimination metrics (area under the receiver operating characteristic curve and area under the precision-recall curve), calibration metrics, and decision-curve analysis; regression performance was evaluated using *R*², mean absolute error, and prediction-interval coverage. A graphical user interface was implemented to enable clinicians to input patient data and obtain calibrated predictions of transfusion probability and corresponding dose recommendations.

**Results:**

MCGB achieved strong discrimination and good calibration across subgroups (area under the receiver operating characteristic curve=0.97 and area under the precision-recall curve=0.91). At a reference probability threshold of .50, sensitivity, specificity, and *F*_1_-scores were 0.99, 0.87, and 0.85, respectively, providing a representative operating point for comparison. For dose prediction among transfused patients, MCGB achieved *R*² of 0.95 and mean absolute error 0.04; 95% prediction-interval coverage was 0.94, indicating accurate point estimates with reliable uncertainty quantification. The software prototype further demonstrated feasibility of real-time decision support at the bedside.

**Conclusions:**

MCGB provides calibrated, interpretable predictions of transfusion need and individualized dose in upper gastrointestinal bleeding and may support bedside decision-making and blood-bank planning, with a prototype interface demonstrating potential for clinical deployment. External validation in additional settings is warranted to confirm generalizability.

## Introduction

Upper gastrointestinal bleeding (UGIB) is a frequent emergency in gastroenterology and acute care [1]. Common etiologies include rupture of esophageal or gastric varices in cirrhosis, peptic ulcer disease, erosive hemorrhagic gastritis, and malignant tumors [2]. Rapid blood loss can precipitate hypotension and coagulopathy with peripheral circulatory failure, progressing to shock and death without timely resuscitation and blood transfusion [3-5]. Although hemoglobin thresholds of 70‐80 g/L are widely cited, clinical application varies across centers and patient profiles [6,7], reflecting differences in comorbidity burden, cardiopulmonary reserve, ongoing hemorrhage risk, and the rate and dose of bleeding [8,9]. These realities motivate decision support that is both individualized and reproducible.

Risk scores such as Glasgow-Blatchford, admission Rockall, and AIMS65 are widely used for early triage in UGIB [10-12]. They stratify short-term risk but do not provide calibrated, individualized probabilities of transfusion or quantitative guidance on transfusion dose. Fixed cutoffs may be sensitive to local practice and case mix and can underperform when clinical heterogeneity is high [13,14]. From a systems perspective, uncertainty about transfusion need and dose complicates blood-bank operations, cross-matching, and inventory management during busy endoscopy schedules or resource-constrained periods. A tool that yields reliable risk estimates and dose guidance could improve bedside decision-making while supporting operational planning.

Machine learning has improved discrimination in many clinical prediction tasks and sometimes outperforms traditional scores [15,16]. However, prior work on transfusion decision support has several limitations that hinder clinical uptake. First, models are often difficult to interpret, which impedes bedside discussion and auditability [17]. Second, probability calibration is inconsistent, limiting safe threshold selection and net-benefit optimization [18,19]. Third, performance can degrade across hospitals and etiologies due to distributional shift. Fourth, preprocessing pipelines sometimes use the full dataset rather than being refit within cross-validation folds, increasing the risk of information leakage and optimistic estimates [20,21]. Finally, most studies stop at binary classification and do not estimate transfusion dose or communicate uncertainty for resource allocation. These gaps suggest that methodological choices should encode clinical directionality, preserve interpretability, control information flow during training, and report both discrimination and calibration alongside clinical utility [22,23].

Electronic health records (EHRs) capture the measurements typically available before a transfusion decision, including demographics, initial vital signs, key laboratory indices, and clinician-adjudicated etiology [24]. Leveraging these data for actionable decision support requires models that map to clinical reasoning while remaining robust across centers [25]. Monotonic constraints can encode established relationships such as higher international normalized ratio (INR) or longer prothrombin time (PT) increasing transfusion risk, and higher hemoglobin, hematocrit, or blood pressure decreasing risk. Curating a small, clinically justified set of feature interactions can improve fit without sacrificing transparency. Calibration methods can align predicted probabilities with observed risks, enabling thresholds derived from decision-curve analysis to reflect clinical utility [26].

This study develops and validates a 2-stage, clinically constrained framework—Medically Constrained Gradient Boosting (MCGB)—for transfusion decision support in UGIB using multicenter EHR data. Stage 1 produces calibrated, interpretable probabilities of transfusion need by imposing monotonic clinical directionality and retaining a stability-screened set of clinically justified interactions. Stage 2 estimates transfusion dose using quantile predictions with conformal adjustment to provide 95% prediction intervals, communicating uncertainty for blood-bank planning. The aim is to deliver a reproducible, well-calibrated, and robust tool that supports bedside decisions and operational workflows across heterogeneous hospitals and etiologies.

## Methods

### Overview

**Figure 1.**
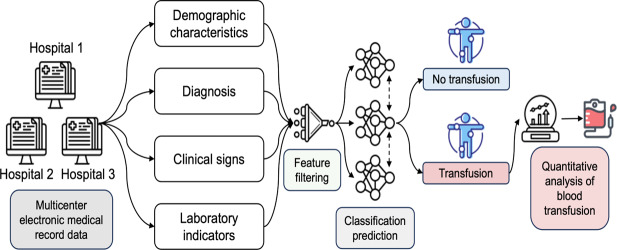
Overall workflow of the MCGB (Medically Constrained Gradient Boosting) framework for transfusion decision support.

The transfusion decision system used a 2-stage framework termed MCGB. A calibrated classifier first estimates the probability of transfusion using monotonic constraints on prespecified laboratory and physiological variables to encode clinically consistent directionality, together with a stability-screened interaction allowlist. To enhance robustness across settings, training optimizes a group distributionally robust objective over hospital, etiology, and admission-period strata; predicted probabilities are calibrated by temperature scaling, and the operating threshold is selected with decision-curve analysis to align classification with clinical utility [27,28]. Patients predicted to require transfusion are then modeled with a regression component that estimates blood units via quantile predictions with conformal adjustment, yielding 95% prediction intervals to communicate uncertainty for blood-allocation planning [29,30]. All preprocessing—Winsorization at the first and 99th percentiles, median imputation, indicator encoding, and interaction construction—is confined to cross-validation folds and applied only to the corresponding calibration and test splits to prevent information leakage. Candidate interactions arise from continuous-by-etiology and continuous-by-continuous pairs, are filtered for high correlation, ranked by mutual information and distance correlation, and retained when they meet prespecified stability criteria, following a screen-in principle that preserves interpretability, reproducibility, and transparency; the overall workflow is shown in Figure 1.

### Ethical Considerations

The study protocol was reviewed and approved by the institutional ethics committees of Daping Hospital, Army Medical Center of PLA, Army Medical University; the Third People’s Hospital of Chongqing; and the 13th People’s Hospital of Chongqing. The requirement for individual informed consent was waived because the analysis used routinely collected EHR data that were deidentified before analysis and posed minimal risk to participants. Data were handled in secure, access-controlled environments under institutional data-use agreements; only coded study identifiers were used, and direct personal identifiers were not accessible to investigators. All procedures complied with the Declaration of Helsinki and applicable local regulations. The project did not involve any prospective intervention and was not registered as a clinical trial.

### Data and Dataset

Routinely collected data were retrieved from the clinical data warehouses of 3 hospitals in Chongqing, China. Consecutive adults with endoscopically confirmed UGIB between January 2019 and August 2025 were screened. A total of 849 patients met the inclusion criteria, including 655 nontransfusion cases and 194 transfusion cases. The cohort comprised 264 patients from the Third Affiliated Hospital of the Third Military Medical University, 326 patients from the Third People’s Hospital of Chongqing, and 259 patients from the 13th People’s Hospital of Chongqing. Exclusion criteria included insufficient clinical data, non-UGIB bleeding, incomplete diagnostic or therapeutic care, self-discharge, transfusion before the index decision, and missing key variables required for model development. The study was approved by the institutional ethics committees of all 3 hospitals, with a waiver of informed consent due to the retrospective design.

Predictors available within 24 hours before the transfusion decision were extracted from the EHR and included demographics, first recorded vital signs at presentation, and initial laboratory results obtained prior to the transfusion decision (covering hematology, coagulation, renal function, albumin, and electrolytes), as well as clinician-adjudicated bleeding etiology encoded as 5 binary indicators. Units were harmonized across centers, and variables not available before the transfusion decision were excluded to prevent data leakage. Patients with missing key variables required for modeling were excluded during cohort construction, resulting in a largely complete analytic dataset. Continuous variables were processed using a median imputation strategy as part of a standardized preprocessing pipeline, although imputation was rarely required in practice due to the near-complete nature of the data. Categorical variables were one-hot encoded, and missingness indicators for selected laboratory tests were constructed to preserve compatibility with potential real-world scenarios in which data may be incomplete. Pairwise correlations were summarized to characterize relationships among predictors while preserving clinically relevant variables. For descriptive comparisons, transfused and nontransfused groups were analyzed using Student *t* tests or nonparametric equivalents for continuous variables and chi-square or Fisher exact tests for categorical variables; 2-sided *P* values are reported in Table 1.

**Table 1.** Baseline characteristics for the nontransfusion and transfusion groups.

Variable	Nontransfusion group (n=655)	Transfusion group (n=194)	*P* value
Demographics			
Male, n (%)	435 (66.4)	126 (64.9)	.67
Female, n (%)	220 (33.6)	68 (35.1)	.67
Age (y), mean (SD)	66.09 (13.74)	66.77 (13.74)	.55
Vital signs, mean (SD)			
Temperature (°C)	36.53 (0.28)	36.58 (0.28)	.03
Pulse (beats/min)	86.61 (14.72)	93.19 (16.85)	<.001
Respiratory rate (breaths/min)	20.03 (2.62)	20.34 (2.62)	.15
SBP^a^ (mm Hg)	123.72 (18.64)	114.37 (20.51)	<.001
DBP^b^ (mm Hg)	72.84 (11.28)	65.07 (12.44)	<.001
Laboratory parameters, mean (SD)			
Hematocrit (%)	36.40 (7.21)	22.57 (6.48)	<.001
Platelets (×10⁹/L)	177.00 (32.40)	187.10 (35.20)	<.001
Hemoglobin (g/L)	105.28 (21.46)	71.72 (18.35)	<.001
Fibrinogen (g/L)	3.09 (0.81)	2.76 (0.77)	<.001
INR^c^	1.19 (0.31)	2.72 (1.16)	<.001
PT^d^ (s)	12.17 (2.18)	17.64 (6.21)	<.001
APTT^e^ (s)	29.39 (5.75)	30.66 (5.75)	.01
BUN^f^ (mmol/L)	10.68 (2.46)	11.58 (2.83)	<.001
Albumin (g/L)	37.91 (5.07)	34.51 (5.45)	<.001
Potassium (mmol/L)	4.13 (0.14)	4.08 (0.16)	<.001
Calcium (mmol/L)	2.26 (0.17)	2.07 (0.19)	<.001
Etiology, n (%)			
REV^g^	141 (21.5)	57 (29.4)	.02
PU^h^	116 (17.7)	91 (46.9)	<.001
AG^i^	116 (17.7)	12 (6.2)	<.001
MT^j^	99 (15.1)	19 (9.8)	.01
Others	183 (27.9)	15 (7.7)	<.001

a SBP: systolic blood pressure.

b DBP: diastolic blood pressure.

c INR: international normalized ratio.

d PT: prothrombin time.

e APTT: activated partial thromboplastin time.

f BUN: blood urea nitrogen.

g REV: rupture of esophageal varices.

h PU: peptic ulcer.

i AG: acute gastritis.

j MT: malignant tumor.

**Figure 2.**
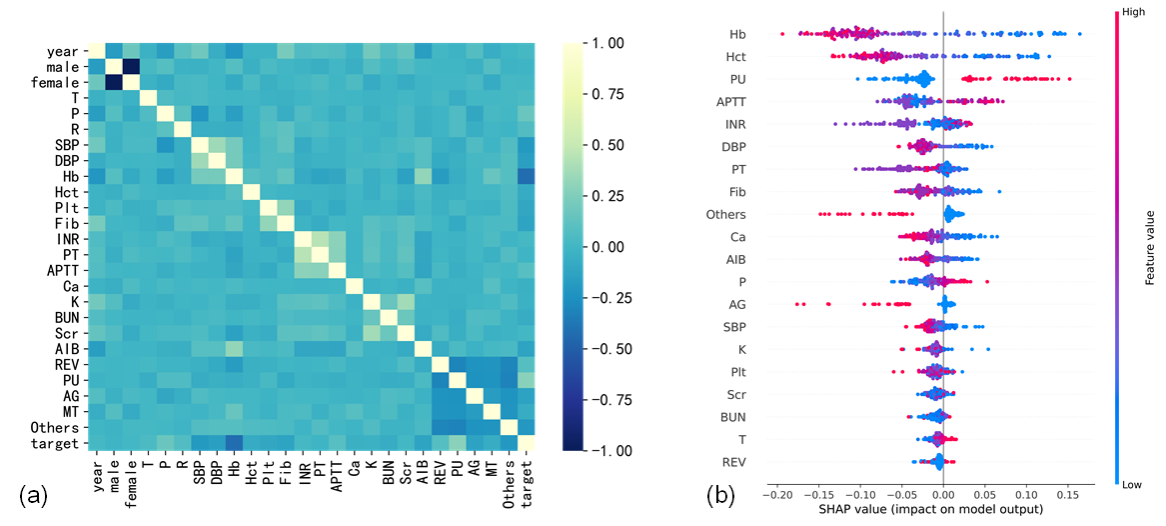
(A) Variable correlation heat map. (B) SHAP summary for transfusion predictors: each dot=patient; x-axis=SHAP value (positive → higher probability, negative → lower); color=feature value (red high, blue low). AG: acute erosive gastritis; Alb: albumin; APTT: activated partial thromboplastin time; BUN: blood urea nitrogen; Ca: calcium; DBP: diastolic blood pressure; Fib: fibrinogen; Hb: hemoglobin; Hct: hematocrit; INR: international normalized ratio; K: potassium; MT: malignant tumor; P: pulse; Plt: platelets; PT: prothrombin time; PU: peptic ulcer; R: respiratory rate; REV: rupture of esophageal varices; SBP: systolic blood pressure; Scr: serum creatinine; SHAP: Shapley additive explanations; T: temperature.

To further characterize the relationships among predictors and enhance transparency in model interpretation, pairwise correlations were first examined using a heat map Figure 2A, providing an overview of feature dependencies while retaining clinically relevant variables. Building on this analysis, model outputs were subsequently examined using Shapley additive explanations (SHAP), as illustrated in Figure 2B. Each point represents an individual patient from the evaluation set, and the horizontal axis indicates the SHAP value, which quantifies the contribution of each feature to the predicted probability of transfusion [31,32]. Positive SHAP values indicate an increased predicted risk, whereas negative values indicate a decreased risk, and the color represents the original feature value, with red denoting higher values and blue denoting lower values. Key predictors showed clinically coherent patterns: lower hemoglobin and hematocrit levels were associated with a higher predicted probability of transfusion, whereas higher values shifted predictions toward lower risk. Coagulation-related indices and hemodynamic variables also exhibited consistent directional effects that aligned with established clinical understanding of bleeding severity and transfusion requirements. Overall, these findings indicate that the model captures clinically meaningful and plausible relationships among predictors, supporting the interpretability and reliability of the MCGB classifier.

### Model Building

In this study, the MCGB framework is proposed to extend standard gradient boosting by integrating 3 clinically motivated components: (1) monotonic constraints to enforce clinically consistent relationships, (2) interaction whitelisting to restrict feature interactions to stable and interpretable pairs, and (3) subgroup-aware reweighting to improve robustness across heterogeneous populations. These extensions are designed to improve both predictive performance and clinical reliability.

The dataset is represented as ${D=(x}_{i},y_{i})$, where $x_{i}\in R^{m}$ denotes the input features, $y_{i}\in R$ denotes the corresponding outcome, and i=1,2,…,n indexes the samples. Following the standard gradient boosting formulation, the boosted predictor after T rounds can be expressed as:


(1) $$\begin{array}{c} \;F_{T}\left(x\right)=\sum_{t=1}^{T}f_{t}\left(x\right),\;\;\;\;\;\;f_{t}\in F,\; \end{array}$$

where F denotes the space of regression trees mapping $R^{m}$ to R. Each tree partitions the data into leaves and assigns a score $w_{j}$. The boosting procedure iteratively refines predictions through stage-wise optimization, while the proposed extensions introduce clinically informed constraints and reweighting mechanisms to enhance robustness and consistency. The optimization objective at iteration *t* is expressed as:


(2) $$\begin{array}{c} Obj_{t}=\sum_{i=1}^{n}l\left(y_{i},\;F_{t-1}\left(x_{i}\right)+f_{t}\left(x_{i}\right)\right)+\gamma T_{t}+\frac{\lambda }{2}\sum_{j=1}^{T_{t}}w_{j}^{2},\; \end{array}$$

where γ controls model complexity by penalizing the number of leaves, and λ is an L2 regularization parameter on leaf weights.

A second-order Taylor expansion is applied around $F_{t-1}\left(x_{i}\right)$. With first- and second-order derivatives defined as$g_{i}=\partial_{\hat{y}}l(y_{i},\hat{y}){|}_{\hat{y}=F_{t-1}(x_{i})}$ and $h_{i}=\partial_{\hat{y}}^{2}l(y_{i},\hat{y}){|}_{\hat{y}=F_{t-1}(x_{i})}$, the approximated loss becomes:


(3) $$\begin{array}{c} l\left(y_{i},\;\;F_{t-1}\left(x_{i}\right)+f_{t}\left(x_{i}\right)\right)\approx l\left(y_{i},\;\;F_{t-1}\left(x_{i}\right)\right)+\;y_{i}\;f_{t}\left(x_{i}\right)+\frac{1}{2}\;h_{i}\;f_{t}{\left(x_{i}\right)}^{2} \end{array}$$

To improve robustness across heterogeneous subgroups, MCGB introduces subgroup-aware reweighting of gradients. Specifically, for each predefined subgroup g, a softmax-based weighting scheme is defined as:


(4) $$\begin{array}{c} \pi_{g}\left(\eta \right)=\frac{\exp\left(\eta L_{g}\right)}{\sum_{\overset{´}{g}}\exp\left(\eta L_{\overset{´}{g}}\right)},\# \end{array}$$

where $L_{g}$ denotes the loss associated with group g, and η§amp;gt;0 is a temperature parameter that controls the sharpness of the weighting distribution. For each sample i, let g(i) denote the group to which the sample belongs. The reweighted derivatives are then defined as ${\tilde{g}}_{i}=\pi_{g\left(i\right)}(\eta )g_{i}$and ${\tilde{h}}_{i}=\pi_{g\left(i\right)}(\eta )h_{i}$, where $g_{i}$ and $h_{i}$ are the original first- and second-order derivatives. This design prevents the model from being dominated by majority subgroups and reduces overfitting to site-specific patterns.

At the tree level, gradients are aggregated within each leaf node. For a leaf *j* with the instance set $I_{j}$, the sufficient statistics are defined as $G_{j}=\sum_{i\in I_{j}}{\tilde{g}}_{i}$ and $H_{j}=\sum_{i\in I_{j}}{\tilde{h}}_{i}$. The quadratic surrogate objective is then approximated as:


(5) $$\begin{array}{c} \tilde{Obj^{\left(t\right)}}=\;\sum_{j=1}^{T_{t}}\left(G_{j}w_{j}+\frac{1}{2}\left(H_{j}+\lambda \right)w_{j}^{2}\right)+\gamma T_{t}.\# \end{array}$$

The unconstrained optimal leaf weight is given by ${\hat{w}}_{j}=-\frac{G_{j}}{H_{j}+\lambda }$. In MCGB, this solution is further adjusted to satisfy clinical monotonicity constraints of the form $\frac{\partial F_{T}(x)}{\partial x_{k}}\sigma_{k}\geq 0$, where k∈M denotes features subject to monotonic constraints and $\sigma_{k}\in \{-1,+1\}$ specifies the expected direction. The feasible solution is obtained by projecting $\hat{w}$onto the isotonic cone:


(6) $$\begin{array}{c} w^{*}=\underset{Aw\leq b}{argmin}\frac{1}{2}||w-\hat{w}|{|}_{2}^{2}, \end{array}$$

ensuring that model predictions follow clinically expected trends and preventing implausible extrapolations, thereby improving generalization and trustworthiness.

The reduction in the surrogate objective when splitting a node into left and right children is quantified by the gain, defined as:


(7) $$\text{Gain}=\frac{1}{2}\left(\frac{G_{L}^{2}}{H_{L}+\lambda }+\frac{G_{R}^{2}}{H_{R}+\lambda }-\frac{(G_{L}+G_{R}{)}^{2}}{H_{L}+H_{R}+\lambda }\right)-\gamma$$

To further enhance interpretability and reproducibility, MCGB restricts candidate splits using an interaction whitelist W. To construct this whitelist, a stability-driven selection procedure is employed. Candidate feature pairs are first evaluated using the Friedman *H*-statistic to quantify interaction strength. Pairs with a median *H*-statistic greater than 0.10 across cross-validation folds are retained. To ensure robustness, only interactions that rank among the top five strongest interactions in at least four out of five folds are selected. This process ensures that retained interactions are both statistically stable and clinically plausible. If a node path already includes feature p, then a split on feature q is admissible only if (p,q)∈W. The whitelist itself is defined by a reproducibility criterion,


(8) $$1\left\{\left(p,\;q\right)\in W=1\left\{median_{r}\;H_{pq}^{\left(r\right)}>0.10\;\wedge Top5_{pq}\;\geq 4\right\}\right\},\#$$

Where $H_{pq}^{\left(r\right)}$ is the Friedman H-statistic in fold r and ${Top5}_{pq}$ is the number of folds in which (p,q) ranks among the 5 strongest interactions. This stability-based selection ensures robustness and reproducibility.

In summary, the MCGB framework extends classical gradient boosting by incorporating 3 clinically motivated components: monotonicity constraints to enforce clinically consistent relationships, interaction whitelisting to restrict feature interactions to stable and interpretable pairs, and group-wise loss reweighting to improve robustness across heterogeneous clinical subgroups. Together, these components enable the model to achieve not only strong predictive performance but also improved interpretability and stability in real-world clinical settings.

To evaluate the effectiveness of the proposed framework, all comparator models were implemented using standard libraries (scikit-learn, extreme gradient boosting [XGBoost], and categorical boosting [CatBoost]) and evaluated under the same cross-site hold-out design as the proposed MCGB model. Within the development cohort, each comparator was trained using the same stratified 5-fold cross-validation splits and fold-specific preprocessing pipeline, and the resulting cross-validation–derived models were subsequently applied to the independent test cohort. This design ensured a fair comparison while maintaining consistent separation between model development and final evaluation. For the classification task, the compared models included logistic regression (LogReg), multilayer perceptron (MLP), random forest (RF), adaptive boosting (AdaBoost), XGBoost [32], and CatBoost [33]; for the regression task, the compared models included linear regression (LR), MLP [34], RF, AdaBoost, XGBoost, and CatBoost. Hyperparameters for all baseline models were tuned via grid search within the training folds only, with model selection based on validation performance.

### Model Training

Model development and validation were conducted using a predefined cross-site hold-out design to assess generalizability. Data from the Third People’s Hospital of Chongqing and the 13th People’s Hospital of Chongqing were combined as the development cohort, whereas data from the Third Affiliated Hospital of the Third Military Medical University were held out as an independent test cohort. The independent test cohort was not used for preprocessing fitting, feature interaction screening, hyperparameter tuning, calibration, threshold selection, or model selection. Within the development cohort, stratified 5-fold cross-validation was used to preserve the transfusion event rate across folds and to perform model development and internal validation. In each fold, preprocessing steps, including median imputation, one-hot encoding, and interaction construction, were fitted exclusively on the training split and then applied to the corresponding validation fold and the independent test cohort, ensuring strict separation and preventing information leakage. Each fold yielded a cross-validation–derived model, and final performance on the independent test cohort was summarized as the mean (SD) across the 5 models. To further examine cross-site robustness, hospital-wise alternating external testing was conducted as a supplementary analysis, in which each hospital was alternately treated as the external test cohort and the remaining 2 hospitals were used for model development. This supplementary analysis was used only to evaluate institutional stability and was not mixed with the primary independent test evaluation.

Given the moderate class imbalance (655 nontransfusion vs 194 transfusion cases), no explicit resampling was performed in order to preserve the original clinical distribution. Model evaluation therefore emphasized metrics robust to class imbalance, including the area under the precision-recall curve (AUPRC) alongside the area under the receiver operating characteristic curve (AUROC). Predicted probabilities were calibrated using temperature scaling within the development cohort, and the learned calibration parameters were then applied unchanged to the independent test cohort. Model performance was primarily assessed across a range of clinically relevant threshold probabilities using decision curve analysis to reflect potential clinical utility. Results at a reference threshold of 0.50 were additionally reported as a conventional operating point for comparison with prior binary prediction studies. For the interaction ablation analysis, net benefit was additionally evaluated at a threshold probability of 0.20 because this value lies within the low-to-moderate risk range in which clinicians may consider early preparation, closer monitoring, or cross-matching rather than immediate transfusion. Therefore, ΔNB@0.20 was used to assess whether the retained interactions improved clinical utility in an early decision-support setting and was not intended to replace the 0.50 reference threshold used for reporting primary classification performance. Primary discrimination metrics included AUROC and AUPRC, whereas secondary metrics included sensitivity, specificity, positive predictive value, negative predictive value, accuracy, *F*_1_-score, and expected calibration error.

For the regression component among transfused patients, predictive accuracy was evaluated using mean absolute error (MAE) and root-mean-square error, explanatory performance using R², and uncertainty using empirical coverage and interval width of 95% prediction intervals derived from conformalized quantile prediction. Agreement between predicted and observed transfusion dose was further assessed using Bland–Altman analysis.

## Results

**Figure 3.**
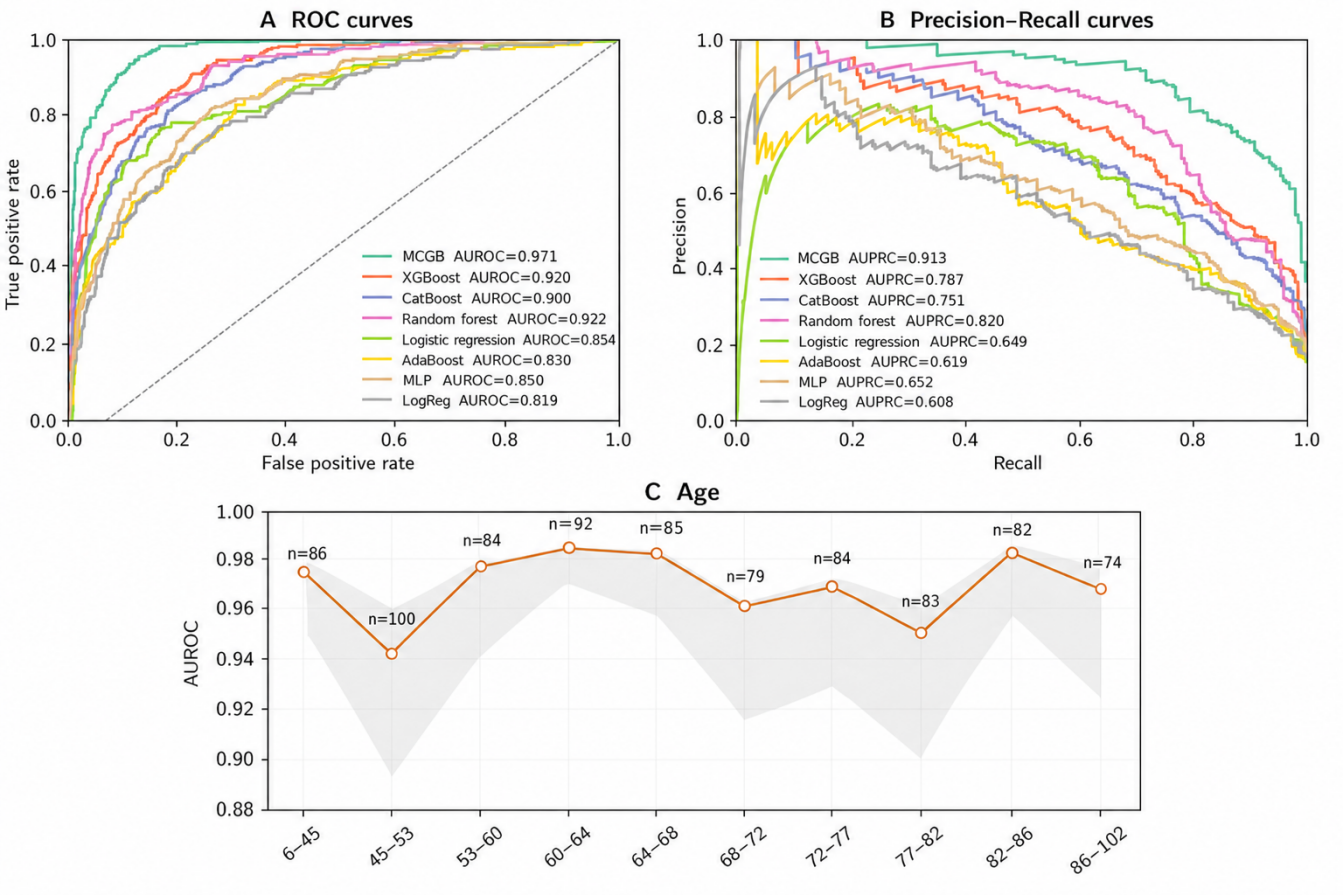
Discrimination and subgroup robustness of MCGB (Medically Constrained Gradient Boosting). (A) ROC curves on the independent test cohort. (B) Precision–recall curves on the independent test cohort. (C) AUROC stratified by hospital in the supplementary hospital-wise alternating external testing analysis. AdaBoost: adaptive boosting; AUPRC: area under the precision-recall curve; AUROC: area under the receiver operating characteristic curve; CatBoost: categorical boosting; MLP: multilayer perceptron; ROC: receiver operating characteristic; XGBoost: extreme gradient boosting.

As shown in Figures 3A and B, MCGB achieved the best discrimination across all models with an AUROC of mean 0.971 (SD 0.008) and an AUPRC of mean 0.913 (SD 0.022). Relative to the strongest baseline, RF, MCGB improved AUROC by 0.049 (0.971 vs 0.922) and AUPRC by 0.093 (0.913 vs 0.820); gains were larger versus XGBoost (ΔAUROC 0.051; ΔAUPRC 0.126) and CatBoost (ΔAUROC 0.071; ΔAUPRC 0.162). The receiver operating characteristic (ROC) and precision–recall curves for MCGB dominate the comparators across clinically relevant thresholds, with the precision–recall separation indicating sustained precision at high recall under class imbalance. For AUROC, the 95% bootstrap intervals do not overlap between MCGB (mean 0.971, SD 0.008) and the leading baselines (RF mean 0.922, SD 0.031; XGBoost mean 0.920, SD 0.020); CatBoost mean 0.900 (SD 0.025), supporting a statistically meaningful improvement. AUPRC intervals are largely separated as well, with only marginal overlap relative to RF.

As shown in Figures 3A and B, MCGB achieved the best discrimination across all models with an AUROC of mean 0.971 (SD 0.008) and an AUPRC of mean 0.913 (SD 0.022). Relative to the strongest baseline, RF, MCGB improved AUROC by 0.049 (0.971 vs 0.922) and AUPRC by 0.093 (0.913 vs 0.820); gains were larger versus XGBoost (ΔAUROC 0.051; ΔAUPRC 0.126) and CatBoost (ΔAUROC 0.071; ΔAUPRC 0.162). The ROC and precision–recall curves for MCGB dominate the comparators across clinically relevant thresholds, with the precision–recall separation indicating sustained precision at high recall under class imbalance. For AUROC, the 95% bootstrap intervals do not overlap between MCGB (mean 0.971, SD 0.008) and the leading baselines (RF mean 0.922, SD 0.031; XGBoost mean 0.920, SD 0.020); CatBoost mean 0.900 (SD 0.025), supporting a statistically meaningful improvement. AUPRC intervals are largely separated as well, with only marginal overlap relative to RF.

Table 2 summarizes classification performance on the independent test cohort. Values are reported as the mean (SD) across the 5 cross-validation–derived models trained within the development cohort. MCGB achieves the best overall performance, with the highest AUROC mean 0.971 (SD 0.008) and AUPRC mean 0.913 (SD 0.022), indicating strong discriminative ability under class imbalance. It also attains the highest accuracy mean 0.922 (SD 0.026) and *F*_1_-score mean 0.847 (SD 0.041), reflecting a favorable balance between precision and recall. Notably, MCGB achieves near-perfect sensitivity mean 0.990 (SD 0.013) while maintaining relatively high specificity mean 0.873 (SD 0.032), suggesting effective identification of patients requiring transfusion with acceptable false-positive rates. Tree-based baselines such as RF and XGBoost show competitive discrimination but consistently lower composite performance, whereas linear and shallow models exhibit reduced recall and *F*_1_-score despite reasonable AUROC. Overall, MCGB provides a more favorable error profile, which is desirable in safety-critical clinical settings.

**Table 2.** Classification performance.

Model	AUROC^a^, mean (SD)	AUPRC^b^, mean (SD)	Accuracy, mean (SD)	Sensitivity, mean (SD)	Specificity, mean (SD)	*F*_1_*-*score, mean (SD)
AdaBoost^c^	0.828 (0.032)	0.617 (0.070)	0.691 (0.028)	0.856 (0.050)	0.642 (0.033)	0.557 (0.044)
CatBoost^d^	0.900 (0.025)	0.751 (0.062)	0.721 (0.033)	0.927 (0.037)	0.661 (0.036)	0.602 (0.051)
LogReg^e^	0.854 (0.031)	0.649 (0.071)	0.855 (0.024)	0.585 (0.068)	0.934 (0.021)	0.647 (0.058)
MLP^f^	0.849 (0.033)	0.652 (0.065)	0.688 (0.030)	0.854 (0.052)	0.639 (0.032)	0.555 (0.049)
RF^g^	0.922 (0.031)	0.820 (0.076)	0.705 (0.031)	0.877 (0.047)	0.654 (0.038)	0.575 (0.047)
XGBoost^h^	0.920 (0.020)	0.787 (0.056)	0.737 (0.029)	0.944 (0.035)	0.676 (0.034)	0.620 (0.047)
MCGB^i^	0.971 (0.008)	0.913 (0.022)	0.922 (0.026)	0.990 (0.013)	0.873 (0.032)	0.847 (0.041)

a AUROC: area under the receiver operating characteristic curve.

b AUPRC: area under the precision-recall curve.

c AdaBoost: adaptive boosting.

d CatBoost: categorical boosting.

e LogReg: logistic regression.

f MLP: multilayer perceptron.

g RF: random forest.

h XGBoost: extreme gradient boosting.

i MCGB: Medically Constrained Gradient Boosting.

Figure 3C presents model performance stratified by hospital, etiology, and age. Across all strata, MCGB maintained consistently high AUROC with relatively narrow 95% CIs estimated via bootstrapping, indicating stable performance despite variation in subgroup sample sizes (eg, n≈264/326/259 across hospitals). Although some strata contained fewer samples, the corresponding CIs did not show substantial widening, suggesting that performance estimates remain reliable. As a supplementary robustness analysis, hospital-wise alternating external testing was conducted by alternately holding out each institution as the external test cohort and using the remaining 2 institutions for model development. This analysis was separate from the primary independent test evaluation and was intended to examine cross-site stability under institutional heterogeneity. In contrast, comparator models exhibited greater variability across strata, with more pronounced performance fluctuations in smaller subgroups. This stability of MCGB is consistent with its design, including monotonic constraints that encode clinically plausible relationships and a curated interaction structure that reduces overfitting to site-specific patterns. Overall, these results indicate that MCGB achieves not only strong discrimination but also robust and consistent performance across demographic and clinical subgroups, supporting its applicability in multicenter settings.

In addition to discrimination and subgroup robustness, the calibration and clinical usability of the proposed MCGB model were further evaluated. As shown in Figure 2, the reliability curves illustrate the agreement between predicted probabilities and observed outcomes across models. MCGB achieves the lowest Brier score, indicating superior overall probabilistic accuracy, although its calibration slope and intercept suggest a degree of overconfidence, which is commonly observed in high-capacity models. To address this, temperature scaling was applied as a post hoc calibration method, resulting in improved probability reliability. Furthermore, decision curve analysis demonstrates that MCGB provides competitive or superior net benefit across most clinically relevant threshold ranges. While RF shows slightly higher net benefit in certain threshold intervals, this difference does not persist across clinically relevant ranges, where MCGB demonstrates consistently strong clinical utility.

**Figure 4.**
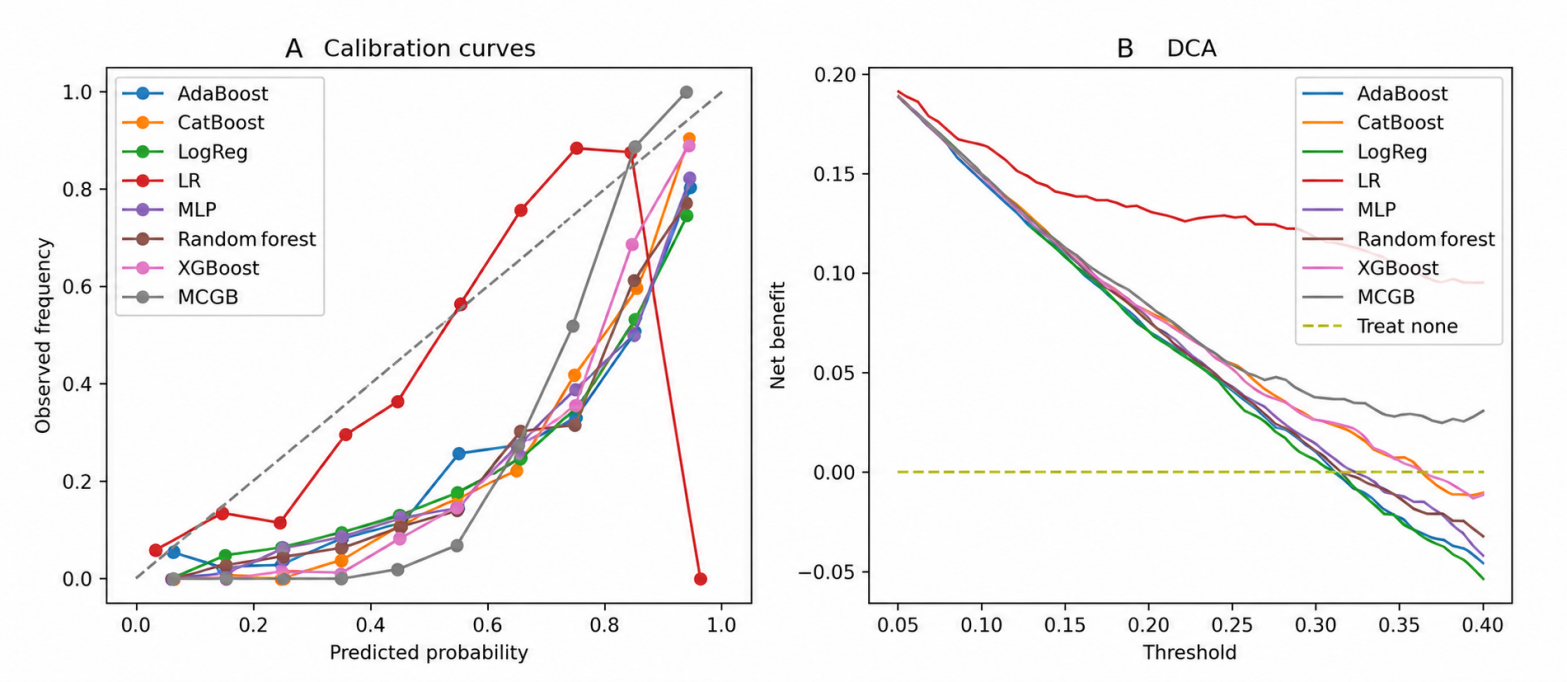
Calibration and decision curve analysis of different models. (A) calibration curves comparing predicted probabilities with observed outcomes. The dashed line represents perfect calibration. (B) Decision curve analysis (DCA) showing the net benefit of each model across threshold probabilities. AdaBoost: adaptive boosting; CatBoost: categorical boosting; DCA: decision curve analysis; LR: linear regression; LogReg: logistic regression; MCGB: Medically Constrained Gradient Boosting; MLP: multilayer perceptron; XGBoost: extreme gradient boosting.

To move beyond aggregate discrimination, we evaluated whether MCGB captures clinically meaningful interactions as shown in Figure 4. A stability-based screen-in rule combined the Friedman *H*-statistic across folds with mean SHAP interaction values and retained reproducible pairs such as INR × PT and systolic blood pressure × pulse, while interactions including blood urea nitrogen × serum creatinine and age × etiology were discarded. Paired ablation on out-of-fold predictions showed reductions in Brier score and gains in net clinical benefit at a decision threshold of 0.20, and the corresponding 95% CIs did not cross zero. Because the interaction analysis was designed to evaluate whether the retained interaction terms improved clinical utility in the early decision-support range, net benefit was assessed at a threshold probability of 0.20. This threshold corresponds to a low-to-moderate predicted risk level at which clinicians may initiate closer monitoring, prepare blood products, or consider cross-matching, whereas the 0.50 threshold was retained as the conventional reference operating point for reporting primary classification performance. Paired ablation on out-of-fold predictions showed reductions in Brier score and gains in net clinical benefit at this threshold, and the corresponding 95% CIs did not cross zero. These findings indicate that MCGB encodes physiologically plausible structure, with INR × PT reflecting coagulation synergy and systolic blood pressure × pulse capturing hemodynamic coupling, which enhances robustness and interpretability beyond overall discrimination.

Among transfused patients, MCGB achieved the lowest prediction error and the highest explanatory power, with a mean MAE of 0.038 (SD 0.016), mean RMSE of 0.099 (SD 0.042), and a mean *R*² of 0.95 (SD 0.024). Because the transfusion dose was normalized during model training, these error values represent deviations on a relative scale; in practical terms, such small deviations indicate that predicted doses are closely aligned with clinician-determined transfusion decisions, typically differing only by a small margin that is unlikely to alter clinical management or blood allocation planning. Compared with the strongest baseline (RF), MCGB substantially reduced MAE (0.128-0.038) and RMSE (0.245-0.099), while improving *R*² (0.88-0.95), with consistent gains across evaluation metrics, suggesting improved predictive accuracy and stability rather than metric-specific improvements. For uncertainty estimation, conformalized quantile prediction achieved a 95% prediction-interval coverage of mean 0.943 (SD 0.021), which is close to the nominal level while maintaining practically useful interval widths; from a clinical perspective, these prediction intervals provide an interpretable range of likely transfusion requirements, supporting communication between clinicians and blood-bank services and facilitating more informed resource planning. In contrast, baseline models showed either under-coverage or less stable interval performance across folds. Overall, these findings indicate that MCGB not only achieves high statistical accuracy but also produces dose estimates that are clinically meaningful, reliable, and directly applicable to transfusion decision-making and operational planning, as summarized in Table 3.

**Table 3.** Dose regression performance across models among transfused patients. Values are reported as mean (SD) across cross-validation folds. MAE^a^ and RMSE^b^ measure the deviation between predicted and actual transfusion dose (normalized scale), *R*² reflects explanatory power, and Coverage at 95% denotes the empirical coverage of 95% prediction intervals.

Model	MAE	RMSE	*R* ^2l^	Coverage at 95%
AdaBoost^c^	0.285 (0.058)	0.373 (0.102)	0.716 (0.041)	0.845 (0.045)
CatBoost^d^	0.262 (0.051)	0.320 (0.094)	0.829 (0.052)	0.867 (0.036)
LR^f^	0.302 (0.049)	0.386 (0.103)	0.670 (0.039)	0.838 (0.048)
MLP^g^	0.247 (0.065)	0.298 (0.098)	0.809 (0.049)	0.918 (0.033)
Random forest	0.128 (0.027)	0.245 (0.086)	0.88 (0.035)	0.912 (0.034)
XGBoost^h^	0.158 (0.036)	0.263 (0.081)	0.85 (0.033)	0.881 (0.032)
MCGB^i^	0.038 (0.016)	0.099 (0.042)	0.95 (0.024)	0.943 (0.021)

a MAE: mean absolute error.

b RMSE: root-mean-square error.

c AdaBoost: adaptive boosting.

d CatBoost: categorical boosting.

e Regression performance.

f LR: linear regression.

g MLP: multilayer perceptron.

h XGBoost: extreme gradient boosting.

i MCGB: Medically Constrained Gradient Boosting.

**Figure 5.**
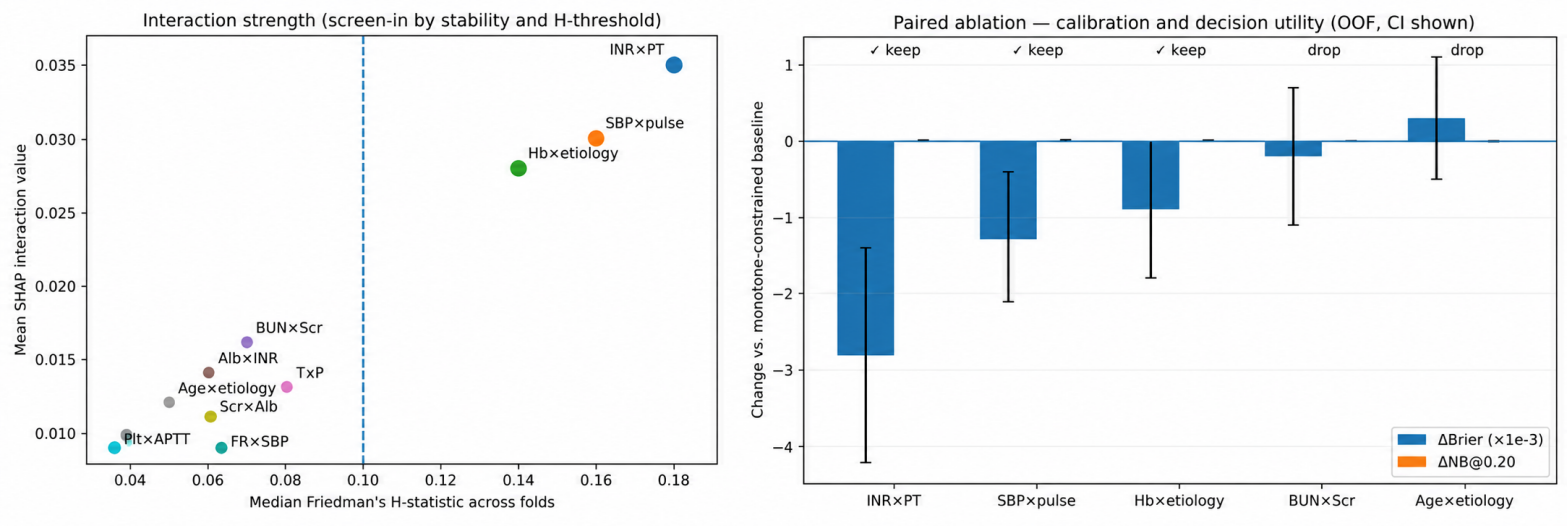
Screening and ablation of feature interactions in MCGB (Medically Constrained Gradient Boosting). (A) Stability-based screen-in rule using the Friedman H-statistic and Shapley additive explanations.

As illustrated in Figure 5, the MCGB regression model demonstrated a close alignment between predicted and true transfusion dose across all 5 cross-validation folds. The predicted curves (blue) consistently tracked the observed transfusion doses (red) with minimal deviation, yielding low MAE and RMSE, alongside a high coefficient of determination. These results indicate that MCGB was able to capture the fine-grained dose-response relationship while maintaining robustness across heterogeneous patient subgroups. The stability of the predictions across folds further suggests that the model generalizes well and avoids overfitting, making it potentially reliable for practical deployment in transfusion planning and blood inventory management.

Comparison with clinical risk scores. To further assess clinical relevance, the proposed MCGB model was compared with established clinical risk scores, including AIMS65, Glasgow-Blatchford Score, and Rockall. As shown in Table 4, these conventional scoring systems demonstrated modest discrimination, with AUROC values ranging from 0.613 to 0.693 and AUPRC from 0.310 to 0.377, whereas MCGB achieved substantially higher performance (AUROC: mean 0.971, SD 0.008; AUPRC: mean 0.913, SD 0.022). Notably, a marked discrepancy between AUROC and AUPRC was observed for the clinical scores. This pattern is consistent with the class imbalance of the cohort and indicates that, although these rule-based scores retain some ability to rank patients by risk, they have limited precision in identifying patients who truly require transfusion, resulting in a higher proportion of false-positive predictions at the individual level. In contrast, MCGB maintained consistently high values across both AUROC and AUPRC, suggesting not only strong global discrimination but also improved precision in detecting clinically relevant cases. This distinction is of particular importance in clinical practice, where AUPRC provides a more informative assessment of a model’s ability to correctly identify patients requiring intervention. The observed differences may be attributed to the design of traditional scores, which rely on a small set of predefined variables and are primarily intended for coarse risk stratification, thereby limiting their capacity to capture complex and nonlinear relationships underlying transfusion decisions. By comparison, MCGB incorporates data-driven modeling with clinically informed constraints, enabling more individualized and reliable predictions. Collectively, these findings suggest that, while established clinical scores remain useful for general risk assessment, they may be insufficient for precise transfusion prediction, and the proposed approach offers potential advantages for supporting clinical decision-making in upper gastrointestinal bleeding.

**Table 4.** Performance of established clinical risk scores for transfusion prediction under stratified five-fold cross-validation.

Model	AUROC^a^	AUPRC^b^
AIMS65	0.613 (0.050)	0.325 (0.062)
GBS^c^	0.693 (0.053)	0.377 (0.060)
Rockall	0.619 (0.045)	0.310 (0.042)
MCGB^d^	0.971 (0.008)	0.913 (0.022)

a AUROC: area under the receiver operating characteristic curve.

b AUPRC: area under the precision-recall curve.

c GBS: Glasgow-Blatchford Score.

d MCGB: Medically Constrained Gradient Boosting.

Through the preceding analyses, the MCGB model demonstrated strong performance in predicting transfusion requirements among patients with UGIB. To facilitate clinical translation, an interactive transfusion recommendation system was developed based on the MCGB model. This system functions as a user-oriented calculator that enables clinicians to estimate the probability of transfusion by inputting routinely available demographic and clinical variables. For patients predicted to require transfusion, the system additionally provides an individualized estimate of the recommended blood dose, thereby supporting both decision-making and treatment planning.

**Figure 6.**
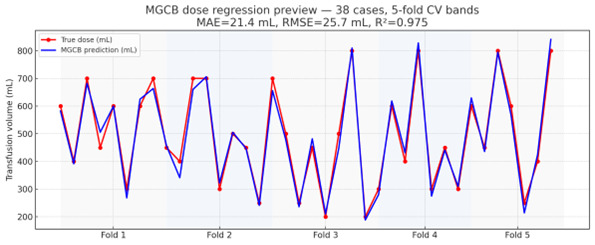
Cross-validation performance of MCGB (Medically Constrained Gradient Boosting) regression model. Comparison of predicted versus true transfusion dose across five cross-validation folds. Red lines denote true transfusion doses and blue lines denote MCGB predictions. Shaded background regions indicate fold partition. CV: cross-validation; MAE: mean absolute error; RMSE: root-mean-square error.

**Figure 7.**
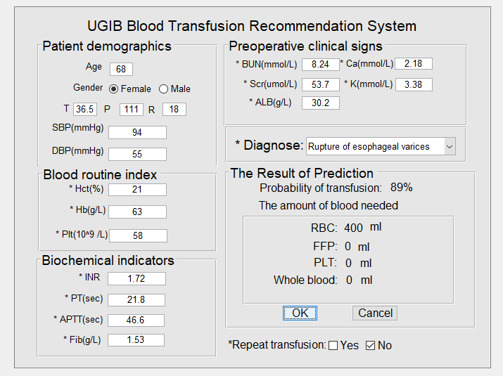
Interface of the UGIB blood transfusion recommendation system. The system integrates patient demographic information, vital signs, laboratory measurements, and clinical diagnosis as inputs, and provides individualized predictions of transfusion probability along with estimated blood component requirements. The interface enables clinicians to obtain real-time decision support for transfusion need assessment and dosage planning, facilitating standardized and interpretable clinical decision-making. ALB: albumin; APPT: activated partial thromboplastin time; BUN: blood urea nitrogen; Ca: calcium; DBP: diastolic blood pressure; FFP: fresh frozen plasma; Fib: fibrinogen; Hb: hemoglobin; Hct: hematocrit; INR: international normalization ratio; K: potassium; Plt: platelet; PT: prothrombin time; RBC: red blood cell; SBP: systolic blood pressure; Scr: serum creatinine.

The graphical user interface, implemented using the QT Designer platform (Trolltech), is designed to be intuitive and easy to operate, allowing real-time interaction within clinical workflows. As illustrated in Figure 6, for a representative patient, the system estimated a transfusion probability of 89% and recommended a red blood cell dose of 400 mL. By integrating predictive modeling into a practical interface, the proposed system provides a feasible pathway for incorporating data-driven decision support into routine clinical practice. In this setting, clinicians can obtain rapid, standardized predictions without additional computational burden, which may contribute to more consistent and timely transfusion decision-making (Figure 7).

## Discussion

### Principal Findings

This study shows that a clinically constrained, 2-stage framework can provide end-to-end decision support for transfusion in UGIB. The classifier delivered very high discrimination together with good calibration, and it maintained performance across hospitals, etiologies, and age strata. These characteristics matter more than a single accuracy number: calibration enables risk thresholds to be interpreted as probabilities, subgroup stability reduces the chance that performance collapses when case mix shifts, and explicit operating thresholds translate model output into consistent actions. In addition, calibration performance is reflected not only in visual agreement but also in quantitative metrics such as expected calibration error and Brier score, which are critical for clinical decision-making [35]. The innovation is not only the use of gradient boosting but the way clinical knowledge is built into the learning process. Monotonic constraints encode clinically expected relationships, and interaction constraints improve interpretability. In the second stage, dose regression provides point estimates with uncertainty, enabling actionable planning rather than binary decisions. In the primary cross-site hold-out evaluation, MCGB maintained strong discrimination and calibration when applied to an independent institutional test cohort.

### Comparison With Prior Work

Traditional UGIB scores are useful for triage but do not provide patient-level probabilities of transfusion or quantitative guidance on dose. Many machine-learning studies report high AUROC yet give little attention to calibration, interpretability, or robustness across sites, and most stop at binary classification [36]. The present work addresses those gaps through several design choices. First, clinical constraints are imposed at training time rather than post hoc, which prevents clinically nonsensical relationships from emerging and reduces the need for manual rule fixes later. Second, interaction curation is principled: interactions such as INR × PT and systolic blood pressure × pulse are retained because they are stable across folds and supported by domain knowledge, and ablation confirms that they improve both calibration and net benefit. Third, evaluation extends beyond global curves to threshold performance, decision-curve analysis, and subgroup reporting, which are the quantities clinicians use when deciding whether and how to act. Finally, the dose module elevates the scope of decision support by estimating the quantity of blood required with explicit uncertainty, which is rarely attempted and directly relevant to operations [37,38].

### Clinical Implications and Workflow

The proposed MCGB framework is designed to support bedside and blood-bank decision-making using routinely collected clinical data. At presentation, the calibrated classifier provides an individualized probability of transfusion need, and a prespecified threshold derived from decision curve analysis encodes the trade-off between missed transfusion and unnecessary cross-matching, enabling standardized triage and reducing subjective variability [39]. For patients exceeding this threshold, the dose model generates both a unit estimate and a 95% prediction interval, supporting clinical communication, cross-matching, and inventory planning [40]. These outputs can be delivered through the interactive UGIB transfusion recommendation system (Figure 6) and integrated into electronic health record systems to enable automated data extraction and real-time risk assessment without disrupting existing workflows. To support safe use, model outputs are intended as decision support rather than definitive recommendations, with probability estimates and uncertainty information provided to assist clinician interpretation and reduce the risk of automation bias. In practice, implementation also requires careful configuration of thresholds and alerts to align with clinical pathways and avoid alert fatigue [41]. Finally, as a clinical decision support tool, MCGB would require prospective validation, external evaluation, and compliance with relevant regulatory frameworks prior to deployment, with clinician oversight and transparent documentation to ensure accountability and safe integration into routine care.

### Limitations and Future Directions

The analysis is retrospective and limited to 3 hospitals within a single regional health care system, which may restrict generalizability to other settings and populations; external and temporal validation is needed to assess transportability and inform recalibration strategies. The dataset is imbalanced with fewer transfusion events, and larger, more balanced cohorts are warranted. Some subgroup strata are relatively small, increasing uncertainty in stratum-specific estimates, although CIs suggest overall stability. In addition, patients with missing key variables were excluded during cohort construction, resulting in model development and validation on a largely complete analytic dataset. While this improves internal consistency, it may not fully reflect real-world deployment conditions, where laboratory measurements may be delayed, unavailable, or selectively ordered at the time of decision-making, requiring explicit strategies for incomplete inputs such as minimum-data requirements or clinician review. Furthermore, thromboelastography and other advanced coagulation indices were not included because they are not routinely available across centers. While such variables may provide additional physiological information, the model was intentionally developed using routinely collected clinical features to enhance generalizability and real-world applicability, and the strong performance observed suggests that commonly available variables capture the majority of clinically relevant signals for transfusion decision-making [42]. Future work should evaluate whether incorporating advanced coagulation markers can further improve performance in settings where such data are available. Although conformal prediction achieved near-nominal coverage, the clinical utility of interval width requires prospective validation. Finally, deployment will require human-in-the-loop safeguards, appropriate alert configuration, and attention to automation bias to ensure safe and effective clinical use.

### Conclusions

This study developed a clinically constrained, 2-stage framework MCGB—to support transfusion decisions in UGIB. By incorporating monotonic constraints that encode established clinical directionality and retaining only stability-screened, clinically plausible interactions, MCGB achieved superior discrimination, reliable calibration, and interpretable predictions. Beyond classification of transfusion need, the framework extends to individualized dose estimation with well-calibrated prediction intervals, offering actionable guidance for both bedside decision-making and blood-bank resource planning. These results demonstrate that MCGB can bridge methodological advances in machine learning with clinical requirements for transparency, robustness, and operational relevance, highlighting its potential as a practical decision-support tool for UGIB management.

## Abbreviations

AdaBoost: adaptive boosting
AUPRC: area under the precision-recall curve
AUROC: area under the receiver operating characteristic curve
CatBoost: categorical boosting
EHR: electronic health record
INR: international normalized ratio
LogReg: logistic regression
LR: linear regression
MAE: mean absolute error
MCGB: Medically Constrained Gradient Boosting
MLP: multilayer perceptron
PT: prothrombin time
RF: random forest
RMSE: root-mean-square error
ROC: receiver operating characteristic
SHAP: Shapley additive explanations
UGIB: upper gastrointestinal bleeding
XGBoost: extreme gradient boosting
